# Cognitive differences between orang-utan species: a test of the cultural intelligence hypothesis

**DOI:** 10.1038/srep30516

**Published:** 2016-07-28

**Authors:** Sofia I. F. Forss, Erik Willems, Josep Call, Carel P. van Schaik

**Affiliations:** 1 Anthropological Institute & Museum, University of Zurich, Switzerland; 2 School of Psychology and Neuroscience, University of St. Andrews, United Kingdom

## Abstract

Cultural species can - or even prefer to - learn their skills from conspecifics. According to the cultural intelligence hypothesis, selection on underlying mechanisms not only improves this social learning ability but also the asocial (individual) learning ability. Thus, species with systematically richer opportunities to socially acquire knowledge and skills should over time evolve to become more intelligent. We experimentally compared the problem-solving ability of Sumatran orang-utans (*Pongo abelii*), which are sociable in the wild, with that of the closely related, but more solitary Bornean orang-utans (*P. pygmaeus*), under the homogeneous environmental conditions provided by zoos. Our results revealed that Sumatrans showed superior innate problem-solving skills to Borneans, and also showed greater inhibition and a more cautious and less rough exploration style. This pattern is consistent with the cultural intelligence hypothesis, which predicts that the more sociable of two sister species experienced stronger selection on cognitive mechanisms underlying learning.

Socially mediated learning^1^ has been studied mainly because it is increasingly shown to be responsible for geographically distinct traditions in many non-human animals^2^,^3^,^4^,^5^,^6^,^7^. However, social learning also turns out to be instrumental in the acquisition of critical ecological^8^,^9^,^10^ and social skills^11^,^12^. The *cultural intelligence hypothesis* proposes that opportunities to learn skills socially during development facilitate the construction of an individual’s intellectual abilities^13^,^14^. Thus, the greater the quantity and quality of such social inputs during ontogeny, the more learned skills an individual can acquire, but also the more experience it can accumulate and thus the better it will be at asocial learning or problem-solving. This process also generates a larger skill pool in a population, which in turn enlarges the individual’s set of learned skills^14^.

So far this developmental dimension of cultural intelligence is well supported both in humans^15^ and nonhuman primates^16^,^17^. However, the hypothesis also has an evolutionary dimension, which posits that species with a social system that predictably exposes maturing individuals to numerous opportunities for social learning will be subject to selection on cognitive abilities, whenever fitness is improved by having a larger set of skills, more complex skills, or mastering them earlier in life. Because maturing individuals in such species will routinely accumulate greater experience, they should be more likely to transfer knowledge to other tasks, and thus further improve their ability to solve problems. This process may select for enhanced exploration strategies. Moreover, on longer time scales, such species should also experience selection to enhance the underlying social learning mechanisms. Importantly, due to the cognitive overlap with asocial learning mechanisms^18^,^19^,^20^, this selection will indirectly also produce improved asocial learning ability, which over time, will lead to an increase in the innate general cognitive performance in conditions identical to the ancestral state, as well as increased brain size.

The cultural intelligence hypothesis should apply to any species that learns socially and transmits this knowledge across generations, although the strength of the effect is likely to depend on the social learning mechanisms, the number of experts, the duration of the learning period, and the role played by experts. The cultural intelligence found in humans can be regarded as an example of this, because the capacity of infants to attend to skills actively demonstrated by experts is an essential ingredient enabling exchange of knowledge across our cooperative and social lifestyle^15^,^21^. Thus, humans have evolved unique predispositions in both infants and caretakers towards active skill transmission (pedagogy: see ref. 22). However, apart from the human case, there are very few formal comparative tests of the correlation between social learning and innovativeness^23^ or brain size as a proxy for asocial learning or innovation ability^24^.

Here we tested the prediction that greater opportunities for social learning are associated with a different exploration style and greater asocial learning ability^14^. A systematic comparison of the cognitive abilities of the two orang-utan species (*Pongo abelii* on Sumatra, and *P. pygmaeus* on Borneo, with an evolutionary divergence estimated from autosomal gene pools of around ~0.9–1.1 Ma^25^) provides an excellent test of this prediction. Orang-utans show extensive social learning during skill development^10^,^26^,^27^, with more frequent peering and subsequent practice as the complexity of foraging skills increases^28^. They also show extensive evidence for geographic variation in a variety of skills^6^,^29^,^30^. Crucially, Sumatran and Bornean orang-utans differ systematically in the frequency of the opportunities for social learning. In similar habitats, Sumatran populations show higher densities^31^,^32^ and are consistently more gregarious and socially tolerant^32^,^33^. They also show much greater repertoires of learned skills and exploratory behaviour^28^, along with greater cultural repertoires in general^6^. This difference in socio-ecology has likely persisted over evolutionary time^25^,^34^. Note that our choice of species provides a particularly stringent test of the hypothesis because their broadly similar brain size^35^ means that we test the genetic impact of cultural intelligence before pronounced brain size differences have evolved.

Because housing and rearing conditions, which may range from deprivation to enculturation, have a major impact on the cognitive development of apes^36^,^37^, a proper test of the possibly subtle differences in cognitive performance crucially requires that the conditions be as identical as possible. Since this cannot possibly be achieved in the natural environment, we therefore turned to zoo-housed orang-utans. All subjects in this study were zoo-born and mother-reared, and experienced highly similar feeding routines, sleeping quarters, encounters with human keepers and visitors, out-door enclosures and enrichment regimes. In all, 33 subjects in 9 different European zoos were tested on their physical cognitive skills on 4–7 different tasks inspired by the test battery employed by Herrmann *et al*.^21^.

When groups of animals differ in cognitive performance, it is informative to look for underlying differences in their problem-solving strategies, because selection on cognitive performance may actually have targeted these mechanisms. We therefore also tested for the possible role of novelty response, exploration style and inhibitory control, since recent literature has identified these as potentially important mechanisms in conspecific comparisons. First, since cognitive tests inevitably involve some element of novelty, how animals respond to novelty may affect their cognitive performance, as found in several studies^23^,^38^,^39^. Second, exploration styles have been reported to influence cognitive performance. Sometimes, the best problem solvers are the boldest individuals^40^, at other times, they are the individuals showing frequent and persistent exploration^41^,^42^, whereas in yet other cases, they are those with the most diverse exploratory actions^43^. Finally, some studies suggest a relationship between inhibitory control and higher cognitive abilities^44^,^45^.

## Results

We presented orang-utans of the two species with a set of physical-cognition tasks and additionally two tests of novelty response (a novel food and a novel toy).

### Cognitive performance

**Figure 1:**
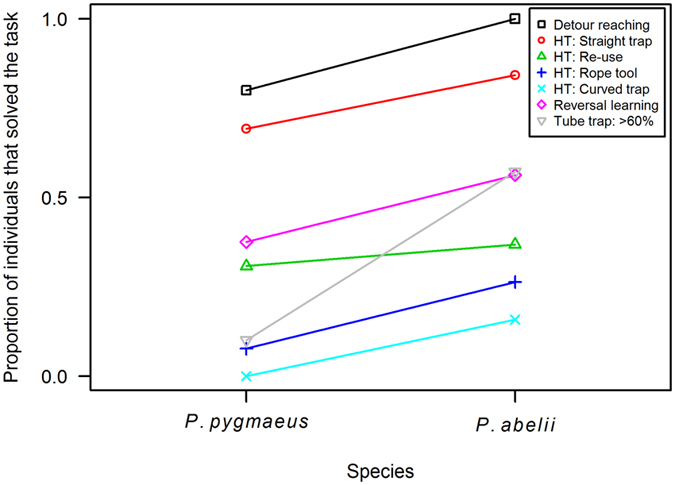
Overall task performance over the different tasks and subtasks by *Pongo pygmaeus* and *Pongo abelii*. Subjects of *P.abelii* were significantly more likely to solve a task than *P. pygmaeus* subjects (Binomial GLMM: B = 1.934, SE = 0.74, z = 2.63, P < 0.01).

Overall performance, across 7 tasks on physical cognition, was assessed for a total of 33 subjects (14 Bornean, 19 Sumatran) from 9 different zoos (Supplementary Table S1 & Table S2). A highly significant binomial GLMM (χ^2^_ML_ = 33.24, N_obs_ = 196, P < 0.0005; Table 1) revealed that, while controlling for the potentially confounding effects of age, sex, group size, the number of zoos a subject had lived in, and task identity, Sumatran subjects were significantly more likely to solve a task than their Bornean congeners (B = 1.934, SE = 0.74, z = 2.63, P < 0.01, Fig. 1, Table 1). In fact, the odds ratio indicated that the overall odds of a Sumatran subject solving a task were more than 6 times as high as those of a Bornean subject. Our model also reflected that, compared to the detour-reaching task, performance was significantly worse on most other tasks (Table 1). Follow-up models constructed to investigate the interaction between species and task, failed to converge due to singularities in the Hessian matrix. However, visual inspection of a plot depicting the proportion of subjects within each species that solved each task (Fig. 1), suggests that the difference between Sumatran and Bornean individuals was both consistent and of a similar magnitude across all tasks (although possibly more pronounced for the tube trap experiment).

**Figure 2:**
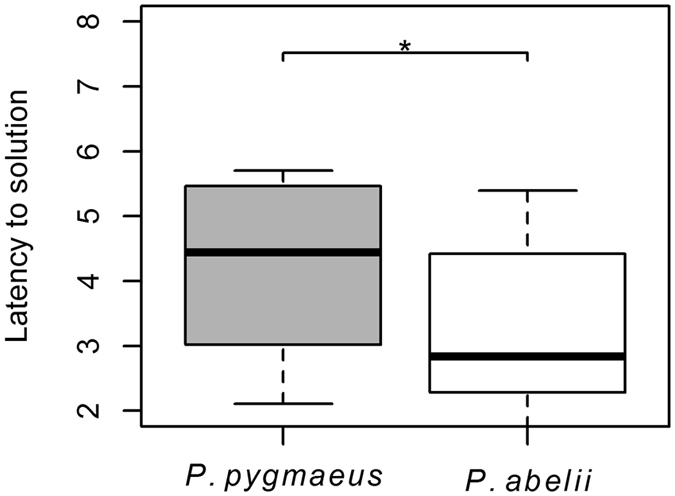
Latency to solution in detour reaching task. Among the subjects who solved the task, Sumatrans showed faster latencies until solution in detour reaching task (LM: N_Sumatra_ = 10, N_Borneo_ = 10, P_species_ = 0.049, β_species_ = −94.53 ± 44.28, P_age_ = 0.155, P_sex_ = 0.760).

The differences between Sumatran and Bornean subjects actually held across individual tasks and subtasks, even though they were not always significant and we could not control for all the confounding variables in these comparisons. The detour-reaching task measured inhibitory control as well as exploration actions (see methods). Data on latency to solution and exploration behaviour was log transformed in order to reach normally distributed residuals. Our results showed that among successful solvers, Sumatran subjects were significantly faster in solving this problem (LM: N_*Sumatra*_ = 10, N_*Borneo*_ = 10, P_species_ = 0.049, β_species_ = −94.53 ± 44.28, P_age_ = 0.155, P_sex_ = 0.760; Fig. 2).

In the honey tool-task consisting of multiple cognitive measurements (see methods), Sumatrans were somewhat more likely to solve the stick solution, but the difference in latencies was not significant (LM: N_*Sumatra*_ = 19, N_*Borneo*_ = 13, P_species_ = 0.159, P_age_ = 0.143, P_sex_ = 0.826). The three individuals who managed to solve the more difficult task of using the rope for the curved trap were all Sumatran.

**Figure 3:**
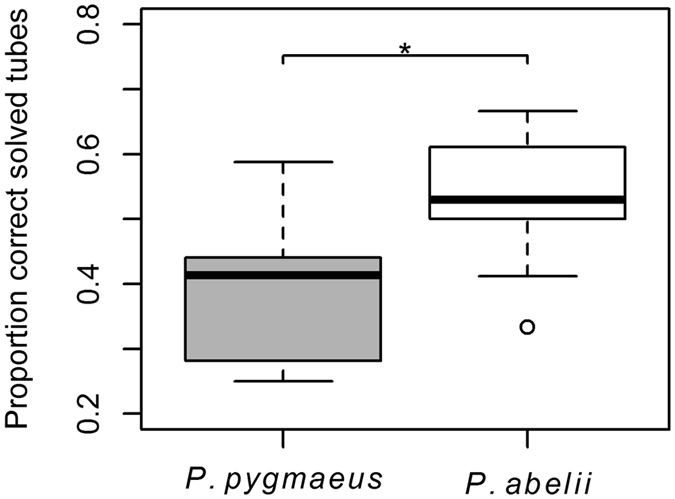
Proportion correctly solved tubes in tube trap task. Sumatran individuals achieved significantly more correct tubes than Borneans (LM: N_Sumatra_ = 14, N_Borneo_ = 8, P_species_ = 0.011, β_species_ = 0.127 ± 0.045, P_age_ = 0.123, P_sex_ = 0.737).

Because the tube trap task (see methods, Supplementary Fig. S1), was designed with equally many tubes providing the correct solution toward the left and the right side, a subject with a strong preference for one side would correctly solve the problem 50 percent of the time. Thus to reach a higher level in this task an individual had to suppress any existing side preference and instead decide in each instant towards which side to move the food item. We therefore first looked for the existence of a side preference and found that Sumatrans and Borneans did not differ significantly in the tendency to have a side preference: 70% for Borneans and 85.7% for Sumatrans (Chi-square test: N_*Sumatra*_ = 14, N_*Borneo*_ = 10, χ^2^ = 2.33, P = 0.311). When comparing the proportion of tubes solved correctly, we found that Sumatran individuals achieved a significantly higher proportion of correct tubes than Borneans (LM: N_*Sumatra*_ = 14, N_*Borneo*_ = 8, P_species_ = 0.011, β_species_ = 0.127 ± 0.045, P_age_ = 0.123, P_sex_ = 0.737; Fig. 3).

In the reversal-learning task all individuals in our sample, both Bornean and Sumatran learnt the first association between lid colour and food reward. Further, 37.5% of the Bornean subjects and 56.3% of Sumatran learnt the reverse colour association (Chi-square test: N_*Sumatra*_ = 16, N_*Borneo*_ = 8, χ^2^ = 0.230, P = 0.891), which did not amount to a significant difference.

### Task exploration

We also examined possible mechanisms that could underlie the species difference in cognitive performance, focusing on the latency to ingest novel food, the exploration during tasks as well as of a novel toy, and an assessment of inhibitory control.

#### Novel food reactions

**Figure 4:**
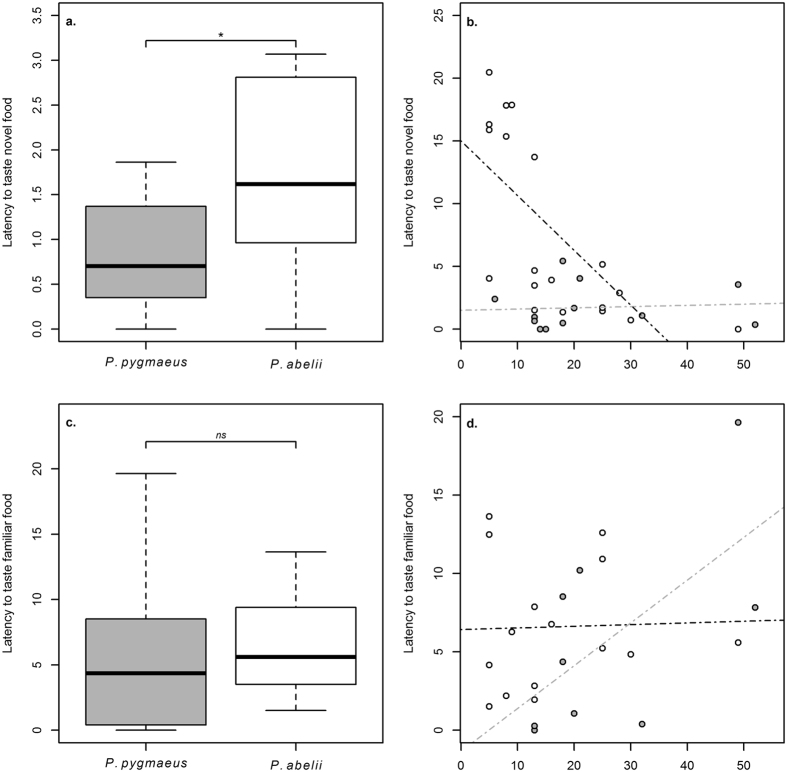
Latencies to taste novel (**a**,**b**) and familiar food (**c**,**d**). Sumatran subjects took longer to taste novel food than Bornean subjects and an interaction effect of age and species was found within the Sumatran subjects: (LM: N_*Sumatra*_ = 14, N_*Borneo*_ = 12, P_species_ < 0.001, β_species_ = 2.123 ± 0.496, P_sex_ = 0.990, P_age_ = 0.781, P_interaction: age/species_ = 0.001, β_interaction: age/species_ = −0.069 ± 0.020). The familiar food condition showed neither a species nor an age effect (LM: N_*Sumatra*_ = 15, N_*Borneo*_ = 9, P_species_ = 0.340, P_sex_ = 0.500, P_age_ = 0.257).

We compared the two species in their response towards novel food, using their reactions to a familiar food item as the control condition. Data of the response variable (latency to taste novel food) was log transferred in order to reach evenly distributed residuals. We found that the Sumatran species took significantly longer before ingesting the new food than Bornean. Moreover, we found an age effect showing that younger Sumatran subjects would take longer to taste novel food than older individuals. However, this age effect was not found in the Bornean sample: (LM: N_*Sumatra*_ = 19, N_*Borneo*_ = 12, P_species_ < 0.001, β_species_ = 2.179 ± 0.433, P_sex_ = 0.726, P_age_ = 0.686, P_interaction: age/species_ = 0.001, β_interaction: age/species_ = −0.071 ± 0.018; Fig. 4a,b). Relative to the Bornean sample, our Sumatran sample contained more young individuals who responded with longer delays to try the novel food. To exclude the fact that those young individuals drove the results of novel food reaction, we also ran the same model excluding all individuals younger than six years. We still obtained the same species difference (LM: N_*Sumatra*_ = 14, N_*Borneo*_ = 12, P_species_ < 0.001, β_species_ = 2.123 ± 0.496, P_sex_ = 0.990, P_age_ = 0.781, P_interaction: age/species_ = 0.001, β_interaction: age/species_ = −0.069 ± 0.020). In the familiar food condition, we found neither a species nor an age effect (LM: N_*Sumatra*_ = 15, N_*Borneo*_ = 9, P_species_ = 0.340, P_sex_ = 0.500, P_age_ = 0.257; Fig. 4c,d).

#### Exploration styles

**Figure 5:**
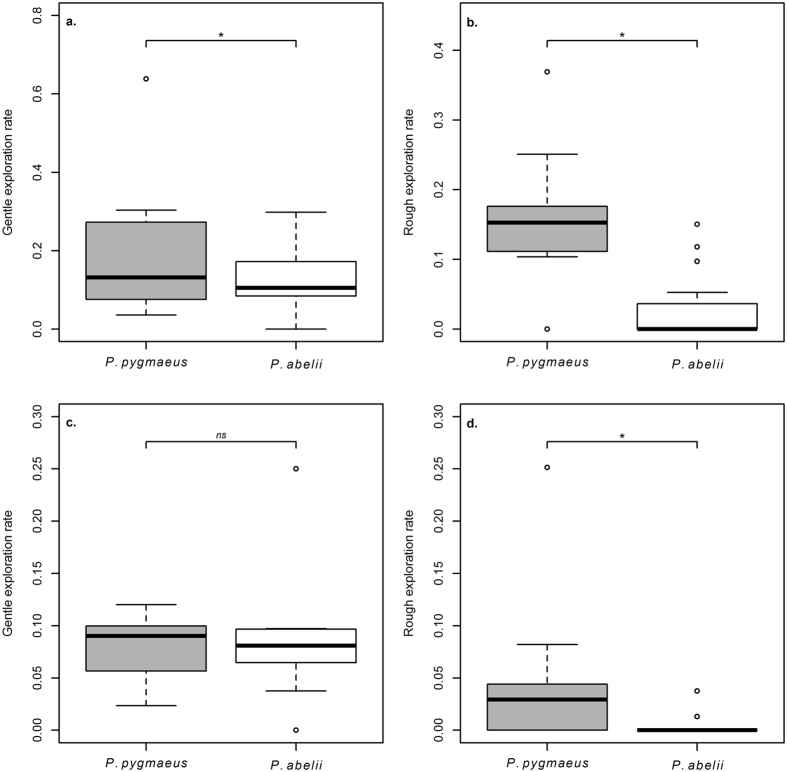
Exploration styles. Exploration rates (corrected for total time at apparatus): gentle exploration and rough exploration for both novel toy (**a**,**b**) (Gentle exploration: LM: N_*Sumatra*_ = 19, N_*Borneo*_ = 9, P_species_ = 0.031, β_species_ = −0.163 ± 0.071, P_age_ = 0.069, P_sex_ = 0.169, rough exploration: LM: N_*Sumatra*_ = 19, N_*Borneo*_ = 9, P_species_ < 0.001, β_species_ = −0.167 ± 0.036, P_age_ = 0.636, P_sex_ = 0.155), and detour reaching task (**c**,**d**) (Gentle exploration: LM: N_*Sumatra*_ = 10, N_*Borneo*_ = 10, P_species_ = 0.648, P_age_ = 0.794, P_sex_ = 0.478, rough exploration: LM: N_*Sumatra*_ = 10, N_*Borneo*_ = 10, P_species_ = 0.042, β_species_ = −0.050 ± 0.023, P_age_ = 0.301, P_sex_ = 0.134).

We also found species differences in the exploration of a novel toy. Compared with Sumatran-, Bornean orang-utans showed a higher rate of gentle exploration, measured as touching, rotating or sliding the tennis balls presented as the novel toy (LM: N_*Sumatra*_ = 19, N_*Borneo*_ = 9, P_species_ = 0.031, β_species_ = −0.163 ± 0.071, P_age_ = 0.069, P_sex_ = 0.169; Fig. 5a) but especially a higher rate of rough exploration, which included hitting, biting or pushing the objects (LM: N_*Sumatra*_ = 19, N_*Borneo*_ = 9, P_species_ < 0.001, β_species_ = −0.167 ± 0.036, P_age_ = 0.636, P_sex_ = 0.155; Fig. 5b).

The same species difference in explorative behaviour found in the novel toy test also appeared in the detour-reaching task. Bornean subjects showed significantly more rough exploration (controlled for time at apparatus) than Sumatrans (LM: N_*Sumatra*_ = 10, N_*Borneo*_ = 10, P_species_ = 0.042, β_species_ = −0.050 ± 0.023, P_age_ = 0.301, P_sex_ = 0.134; Fig. 5d). We did not find the same effect when comparing gentle exploration (LM: N_*Sumatra*_ = 10, N_*Borneo*_ = 10, P_species_ = 0.648, P_age_ = 0.794, P_sex_ = 0.478; Fig. 5c).

**Figure 6:**
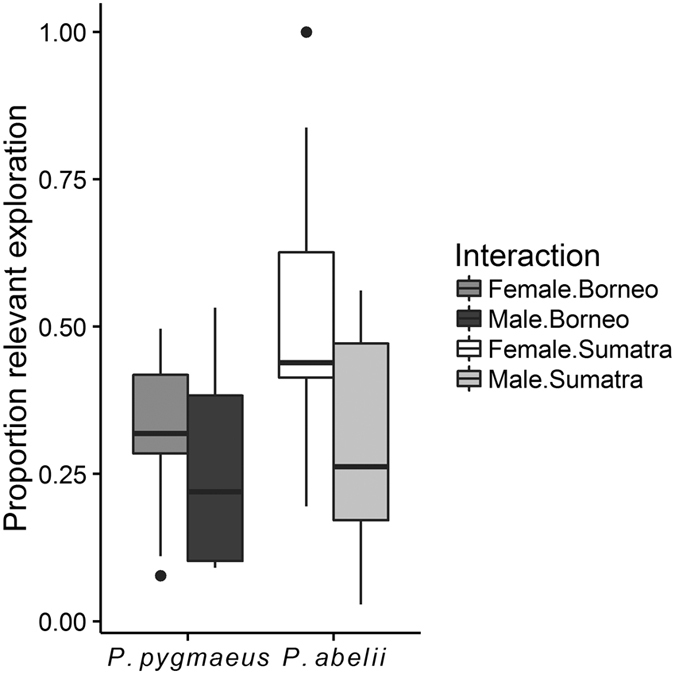
Relevant exploration. Proportion of relevant exploration time devoted to the honey extraction, corrected for total exploration duration of apparatus. Sumatran females spent more time exploring the relevant parts of the problem solving apparatus than Bornean, and males showed less relevant exploration time than females (LM: N_*Sumatra*_ = 19, N_*Borneo*_ = 13, P_species_ = 0.064, β_species_ = 0.139 ± 0.072, P_age_ = 0.210, P_sex_ = 0.029, β_sex_ = −0.183 ± 0.080).

In the honey tool-task, each subject was given ten minutes to engage with the apparatus in order to extract honey using the correct tool for two different traps; straight and curved trap. There was no species difference in either the attentive time (LM: N_*Sumatra*_ = 19, N_*Borneo*_ = 13, P_species_ = 0.903, P_age_ = 0.064, P_sex_ = 0.811, Supplementary Fig. S2a), or the duration of exploration in this task (LM: N_*Sumatra*_ = 19, N_*Borneo*_ = 13, P_species_ = 0.398, P_age_ = 0.094, P_sex_ = 0.449, Supplementary Fig. S2b), indicating that individuals of both species were equally motivated to engage with the task. They also did not differ in the variety of exploration actions (LM: N_*Sumatra*_ = 19, N_*Borneo*_ = 13, P_species_ = 0.930, P_age_ = 0.465, P_sex_ = 0.523, Supplementary Fig. S2c). However, we found that Sumatrans clearly tended to spend more time exploring the relevant parts of the problem-solving apparatus compared to Borneans, and that males spent less time on relevant exploration than females (LM: N_*Sumatra*_ = 19, N_*Borneo*_ = 13, P_species_ = 0.064, β_species_ = 0.139 ± 0.072, P_age_ = 0.210, P_sex_ = 0.029, β_sex_ = −0.183 ± 0.080; Fig. 6).

#### Inhibition

**Figure 7:**
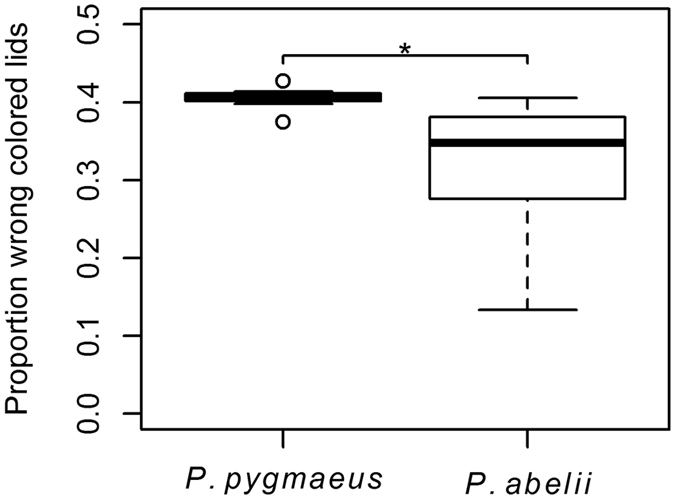
Reversal learning task. Proportion of lids opened of wrong colour corrected for total amount of lids touched in the reversal learning task. Sumatran subjects opened less wrong coloured lids than Bornean subjects, (LM: N_*Sumatra*_ = 16, N_*Borneo*_ = 8, P_species_ = 0.011, β_species_ = −0.092 ± 0.033, P_age_ = 0.899, P_sex_ = 0.475).

The reversal-learning task provides the opportunity to examine inhibition. When an individual opens the correct lids it acquires information about the specific colour and presence of a food reward; equally, opening the wrong lids produces information about the absence of a food reward associated with that colour. Once the individual has learned where the food is hidden it should therefore inhibit the tendency to open the wrong lids. We calculated the total number of lids each subject touched and the proportion of which were of the wrong colour and log transferred our data to produce evenly distributed residuals. We found a significant difference between the species: Bornean orang-utans opened more of the wrong coloured lids than did Sumatran, (LM: N_*Sumatra*_ = 16, N_*Borneo*_ = 8, P_species_ = 0.011, β_species_ = −0.092 ± 0.033, P_age_ = 0.899, P_sex_ = 0.475; Fig. 7).

## Discussion

The results showed a clear and consistent pattern: Sumatran orang-utans, *Pongo abelii,* performed better in a variety of tests of physical cognition compared to the Bornean species, *Pongo pygmaeus* (Fig. 1 and Table 1). In fact, there was not a single task in which Bornean subjects were more likely to solve the problem than the Sumatrans. The results of the GLMM reveal that variation in cognitive performance was strongly determined by species and revealed no significant effect of group size, age, sex, or the identity of the zoo in which they were kept. Moreover, in the detour-reaching task Sumatran orang-utans were faster at achieving the solution, which required inhibition of fixation on the visible food reward (Fig. 2). In the tube-trap task no subject manage to solve more than 12 tubes out of 18, perhaps because most individuals had a side preference, which would have to be suppressed in order to reach a high task performance. Nonetheless, Sumatran orang-utans managed to solve more tubes correctly than the Borneans (Fig. 3). These results therefore support the existence of an intrinsic species difference in the ability to solve physical cognition tasks, in agreement with the prediction of the evolutionary version of the cultural intelligence hypothesis.

Given this clear difference in performance on tasks of physical cognition between these two closely related species, it is of great interest to identify possible underlying variables. We measured novelty response, inhibition and aspects of exploratory behaviour. Because Sumatrans were more cautious in tasting novel food (Fig. 4), better performance was not due to reduced neophobia, as was found in some previous studies^38^,^41^. The species difference was also not confounded by age effects. Although younger Sumatrans delayed their intake of novel food, when tested individually (Fig. 4b), the species difference remained even when we excluded the younger subjects from the Sumatran sample. Species with greater dependence on social learning have been suggested to also exhibit higher neophobia and conservative novelty response, because they strongly rely on social cues to engage in independent exploration^46^. However, captivity has been shown to suppress neophobia in orang-utans^47^. Therefore, it is remarkable that we still detect this species difference in a zoo comparison, suggesting a stronger predisposition for cautiousness in Sumatran orang-utans than Borneans.

In the honey tool-task, which consisted of multiple problem-solving steps, both species were equally keen on participating and spent equal time exploring the task (Supplementary Fig. S2), but Sumatran females, though not males, spent more time exploring the relevant parts of the apparatus (the holes containing honey; Fig. 6). Further, Bornean orang-utans were more likely to apply a rougher exploration style than their Sumatran relatives, both in the detour-reaching box and toward the novel toy (Fig. 5b,d). Such rough actions suggest that the subjects had given up on trying to find a solution and were either frustrated or attempting to reach the food reward through force, or both.

Reversal-learning tasks entail an element of inhibition^48^. A higher percentage of Sumatran individuals learned the colour reversal. Although this was not significant, we found that Sumatrans were also better at inhibiting their behaviour in that they opened significantly fewer lids of the wrong colour than Borneans (Fig. 7). Orang-utans have previously been reported to exhibit higher inhibitory control than other great apes^45^, but our results suggest that Sumatrans show this even more than the Borneans, which complements their greater cautiousness and gentler explorative behaviour.

In sum, the superior cognitive performance by the Sumatran orang-utans may well reflect their greater inhibitory control and more cautious exploration style, which made them less likely to turn to destructive exploration and more likely to focus on relevant aspects of the problem at hand.

Because group size in the zoos examined did not affect the results and the zoos did not differ greatly in their enrichment regimes, this species difference cannot reflect any differences in opportunities for social or asocial learning during development other than those caused by innate differences in attention patterns or social tolerance by role models. Moreover, it is unlikely to be due to innate differences in the ability to effectively manipulate tools, because on Sumatra the tendency to use tools is limited to particular regions^32^. Zoo orang-utans of both species use tools regularly and all nine zoos where the data was collected provided the apes with enrichment devices requiring stick tool-use, with which all subjects in our study were familiar.

If the species had been very different in overall or relative brain size, the same result would presumably have been obtained, given the effect of brain size on cognitive abilities in primates^49^,^50^ and carnivores^51^. The study was designed to capture the effects of cultural intelligence at similar brain size. Nonetheless, there are minor brain size differences between the two orang-utan species. Although females are all approximately the same body size, those of the two western Bornean subspecies have a cranial capacity that is slightly (average 2–3%) smaller than that of the Sumatrans. However, those of the eastern subspecies *Pongo pygmaeus morio* have a cranial capacity that is on average 11–12% smaller than that of the other Bornean subspecies and 14% smaller than that of the Sumatrans^35^. However, because the breeding program in European Zoos that manages the population of *Pongo pygmaeus*, does not distinguish between subspecies, we do not know which individuals, if any, are of this subspecies, assuming there are any pure or hybrid *P. p. morio* at all in European zoos. Moreover, the brain size distributions between the species and subspecies show high overlap, and any difference in relative brain size is still less than that between the sexes of modern humans^52^. Furthermore, the greater interspecific variation in absolute and relative brain size among all great apes, relative to that found between Bornean and Sumatran orang-utans, does not translate into consistent differences in quantitative reasoning or inferential reasoning^53^,^54^. Most importantly, however, regardless of any residual effects of brain size, we identified plausible underlying causal differences in exploration style, which help us understand the species differences found here and may also be involved in species differences across a broader range of brain sizes (e.g. inhibitory control^55^).

This species comparison of physical cognitive tasks provides the first empirical confirmation of the cultural intelligence hypothesis in a non-human species, suggesting that the combination of more frequent opportunities for social learning and advanced skill repertoires have over evolutionary time produced cognitive differences between the two *Pongo* species. More generally, the traditional benefit hypotheses for the evolution of intelligence, such as the social brain hypothesis^56^,^57^ or the technical intelligence hypothesis^58^, both face the problem of grade shifts, i.e. that different lineages show major differences in intelligence in spite of similar social or technical challenges^24^,^59^. The cultural intelligence hypothesis, which basically argues that where learning is more efficient intelligence can be enhanced, may therefore be essential to complement the explanatory power of these benefit hypotheses.

## Methods

### Subjects

We undertook the study in nine European zoos (Table S1, supplementary material), where both species of orang-utans are housed under constant and similar conditions, and tested only mother-reared individuals. The European breeding program, EEP, holds all detailed information on birth dates, kinship, transfers and island of origin of all orang-utans in European zoos. Supplementary Table S1 describes the housing conditions and the time at which the experiments were run at each zoo.

During the cognitive tasks all subjects participated on their own initiative and individually, which controlled for variation in motivational state between subjects, albeit at the expense of a reduced sample size in some tasks. All tests were conducted either in the morning hours or around mid-day and all subjects were fed normally before and after participating in the tasks. The tasks were presented to the orang-utans in their smaller sleeping enclosures or directly in the large home enclosure, whenever a subject could be separated from the rest of the group there. If mothers could not be separated from their dependent offspring, they were tested together with their infants (the latter did not participate in the tasks). Participating subjects ranged in age from five to fifty-two years (Table S2, supplementary material). The average age was 17 years for Sumatran subjects, 21 years for Borneans. All tasks were video recorded with two SONY HDR-CX200 Handy cameras, because no humans were nearby or interacting with the subject during testing so as to minimize human impacts.

### Ethical note

All experiments were purely behavioural and fully complied with the ethical guidelines of each zoo, the European Directive 2010/63/EU, and were approved by the ethics committee of the University of Zurich in Switzerland. Further, all data collected in the United Kingdom were approved by the British and Irish association for zoos and aquariums, BIAZA.

### Description of physical cognition tasks

Inspired by the primate cognition test battery (PCTB) of Herrmann *et al*.^21^ we developed a modified set of physical cognition tasks to assess different aspects of cognition. These tasks were modified because we wanted to make it possible to collect meaningful information without pre-training and frequent interactions with humans, and therefore had to make them as naturalistic and simple as possible, as well as adjust them to different locations of testing.

#### Detour reaching task

A large transparent plexiglas box (100 cm × 30 cm × 30 cm) was presented in the sleeping enclosure of the subjects. Because the box was placed inside the enclosure it was entirely accessible to the subjects to explore the whole box, providing suitable measurements of explorative actions. Exploratory actions of the plexiglas box were divided into two categories: rough (push, pull, hit) and gentle (touch, poke). The front side of the box had two openings, one small round hole (diameter 2 cm) and one large rectangular opening (30 cm × 20 cm) situated 50 cm from the small opening (Supplementary Fig. S1a). Before the subject entered the test enclosure a food reward (piece of fruit) was placed inside the plexiglas box right behind the small opening, through which the food reward did not fit. The subject would have to prevent its focus on the visible fruit in order to find the large opening and thereby the solution. Each subject was given five minutes to solve this task and the task started as soon as the subject approached to within one meter from the box. The moment the subject touched the fruit piece inside the box was counted as a successful solution and ended the task.

#### The honey tool-task

This problem-solving task presented a wooden box (50 cm × 80 cm × 5 cm) with two traps, which were covered with a plexiglas on the front side for visibility (Supplementary Fig. S1b). The upper trap was a straight, downward-sloping channel (30 cm × 5 cm) filled partly with honey, in which a 40-cm long stick was already inserted (and thus immersed into the honey). The second, lower trap was an L-shaped curve (15 cm × 10 cm), whose interior part, also filled with honey, could not be reached with a finger or a stick. We additionally provided two sticks (40 cm) and three bendable plastic ropes (20 cm) on the floor in front of the apparatus. In order to find the solution for the L-shaped trap, the subject needed to use one of the provided ropes as a tool and dip it into the L-shaped trap. The rope could also be explored as a tool in the straight trap but did not yield any honey reward due to its insufficient length. Likewise, the stick could not reach the honey in the L-shaped trap. The total time a subject was given for this task was ten minutes.

First, we assessed how attentive subjects were toward the test apparatus by calculating the time they spent within one meter of the apparatus as well as the duration of exploration of the apparatus. Exploration was defined as any event were the subject would touch and manipulate any part of the apparatus or the different tools provided right beside the apparatus, minus the time that was spent at performing the solution, e.g. dipping the stick into the straight trap. We also recorded relevant exploration events, which included all exploration events directed toward the two traps and not the apparatus itself (and thus relevant to the actual problem-solving). From this, we calculated the proportion of total exploration duration during which the subjects focused on relevant exploration. Second, for the cognitive performance we used four measurements from this task:

- Use of the information provided beforehand: re-use of the stick that was already provided as solution in the straight trap. The stick was counted as re-used if the subject did not let go of it, walked out of sight with it or put it on the floor before re-inserting it into the straight trap. A stick was defined as inserted if at least one third of the stick was inside the straight trap.
- Correct solution to the straight trap: if the subject did at any point during the ten minutes insert the stick to the straight trap, it was defined as a successful solution to the straight trap.
- Considering the rope as a tool: if the subject did at any point during the ten minutes tried the rope as a tool for either of the traps.
- Correct solution to the L-shaped trap: if the subject inserted the rope tool into the L-shaped trap during the ten minutes. Any act where a subject inserted the rope and thus recognized that the rope was the correct tool for the L-shaped trap was regarded correct solution, regardless of whether the subject actually obtained any honey.

#### The tube-trap task

The tube-trap task was also presented to each subject outside of the enclosure mesh, along with sticks to reach six horizontal metal tubes (Supplementary Fig. S1d). Each tube was 30 cm long and 5 cm wide, with an opening on either end, where the stick could be inserted to slide a visible reward (a piece of fruit or a nut) in two different directions. However, the tube had a trap, visible from the outside. Thus, if the reward was moved in the wrong direction it would fall down a 10 cm deep metal cylinder and get trapped. However, if the reward was moved in the correct direction it reached the end of the tube and fell out, to be picked up. The board contained six tubes. Each subject encountered the task in three consecutive trials, resulting in 18 possible attempts. Three tubes had the correct opening on the left side, three on the right side. Thus, if a subject would have a strong side preference and always slid the reward toward one side, it would reach nine correct out of 18 (50%). We therefore calculated the percentage of tubes an individual solved correctly and used a criterion of more than 60% of the tubes correct as a successfully solved task.

#### Reversal learning task

In this task the orang-utans were presented a wooden board, at a distance of ca. 20 cm outside of the enclosure mesh (Supplementary Fig. S1c). The board had 12 holes with 12 lids: six black and six white ones. The subjects were provided sticks to reach the lids of the board. In the first part of the task a food reward (fruit piece or nuts, depending on recommendations or preference of the keepers) was hidden behind either all the black or all the white lids (colour was randomly determined for each subject). We determined that the subject had successfully learned the association between right colour lid and food reward once at least five out of the six first lids it touched were of the correct (rewarded) colour. In addition, the subject had to pass an extra control trial to ensure it had learned the right association. After the control trial was also successful, we switched the position of the food reward to the opposite colour, and counted if and how many trials it took the subject to learn the reverse pattern. The task continued for four days and each subject was given three to four trials per day (depending on when a control trial was needed or not).

### Novelty response tests

#### Novel food

As a novel food item we used potato mash that was coloured turquoise using regular food colouring and topped with a few black olives (Supplementary Fig. S1e). Each subject was then served a handful of the turquoise potato mash as a little pile on a board right outside the mesh of the test location. The novel food test lasted for a maximum of two minutes, but ended earlier in case all food had already been consumed. We measured the latency to taste the novel food as a proxy for cautiousness. We used the latency of tasting from the point when the subject first touched the item to control for potential differences due to the size of the enclosure mesh through which the subjects had to reach for the food items. As a control condition we also recorded reactions toward a familiar food item, which was either a fruit or vegetable that was part of the subjects’ daily diet.

#### Novel toy

As a novel toy we presented the orang-utans with a wooden board containing three slits, in each of which sat two differently coloured tennis balls that could be rotated and moved in different directions (Supplementary Fig. S1f). Subjects were given two minutes to interact with the novel toy. Since many zoo-housed orang-utans are familiar with tennis balls (albeit not with these colours or in this context), our intention for this task was to capture how they explore a new task that neither presents any particular problem to be solved nor produces a food reward. Explorative behaviour of the toy was divided into the same categories as for the detour-reaching task: rough- (bite, hit, pull, push) and gentle exploration (touch, poke, rotate, slide). We calculated exploration rates, counted as number of total exploration events of each category divided by the total time spent with the toy.

### Statistical Analyses

The same observer (SF) coded all behaviour details from the videos of each task using Mangold Interact 9.7. The sample size for each task varied somewhat, because zoos differed slightly in opportunities for separate testing and not all individuals could always be separated. To test for a potential species difference in overall performance (task solved: yes/no), we fitted a Generalized Linear Mixed-effects Model (GLMM) with a binomial error distribution to the data. We incorporated species as the main fixed effect, while task identity, age, sex, group size, and the number of zoos the subject had lived in over the course of its life-time, were included as additional (confounding) fixed effects. Planned contrasts for task (the only categorical predictor with more than two levels) were set to compare a subject’s performance on each task to its performance on the detour-reaching task (i.e. the task with the highest overall performance, solved by all but 2 subjects). We controlled for repeated observations on each task across the same subjects from different zoos by specifying task identity and individual identity nested within zoo as two crossed random effects. For the exploration data of each task (time to solution) we used standard linear models, with species as independent variable while controlling for age and sex. All statistical analyses were conducted in R version 3.2.3, using the “lme4” package^60^.

## Additional Information

**How to cite this article**: Forss, S. I. F. *et al*. Cognitive differences between orang-utan species: a test of the cultural intelligence hypothesis. *Sci. Rep.* **6**, 30516; doi: 10.1038/srep30516 (2016).

## Supplementary Material

Supplementary Information

## Figures and Tables

**Table 1:** Output from the generalized linear mixed model (GLMM) showing species differences in task performance.

	B	SE	z value	*P*
Intercept	1.998	1.44		
Species				
* Pongo pygmaeus*	–	–	–	–
* Pongo abelii*	1.934	0.74	2.63	0.0085
Confounding variables:				
Task				
* Detour reaching*	–	–	–	–
* HT: Straight trap*	−1.446	0.97	−1.49	0.1355
* HT: Re-use*	−4.147	1.06	−3.92	0.0001
* HT: Rope tool*	−5.258	1.15	−4.58	0.0000
* HT: Curved trap*	−6.268	1.27	−4.93	0.0000
* Reversal learning*	−3.441	1.06	−3.26	0.0011
* Tube trap: >60%*	−4.260	1.12	−3.81	0.0001
Sex				
* Female*	–	–	–	–
* Male*	0.448	0.72	0.63	0.5321
Age	0.006	0.03	0.21	0.8306
Number of Zoos	−0.543	0.31	−1.77	0.0774
Group size	0.122	0.12	0.99	0.3235

196 observations on 33 individuals from 9 different zoos, χ^2^_ML_ = 86.45, *P* < 0.0001.
