# Supplementary material for: Cognitive differences between orang-utan species: a test of the cultural intelligence hypothesis
Source: Sci Rep. 2016 Jul 28;6:30516. doi: 10.1038/srep30516 (PMC4964338; doi:10.1038/srep30516)
Supplement: Supplementary Information [file srep30516-s1.pdf]

Cognitive differences between orang-utan species: a test of the cultural intelligence hypothesis

Sofia I. F. Forss[1], Erik Willems[1], Josep Call[2] and Carel P. van Schaik[1]

[1]*Anthropological Institute & Museum, University of Zurich, Switzerland*

[2]*School of Psychology and Neuroscience, University of St. Andrews, United Kingdom*

Supplementary material:

Supplementary Table S1: Zoos where data was collected.

Zoo	Species	Time of study	Group size	Outdoor Enclosure	Sleeping quarters	# permanent enrichment devices
Twycross zoo	*Pongo pygmaeus*	January-February 2013	4	Yes	Yes	1
Allwetter zoo münster	*Pongo pygmaeus*	April- May 2013	6	Yes	Yes	3
Apenheul Primate Park	*Pongo pygmaeus*	January-February 2014	12	Yes	Yes	2
Blackpool zoo	*Pongo pygmaeus*	January 2015	4	Yes	Yes	1
Paignton zoo	*Pongo pygmaeus*	February 2015	5	Yes	Yes	1
Dortmund zoo	*Pongo abelii*	November-December 2012	6	No	Yes	1
Durrell Wildlife Trust	*Pongo abelii*	March 2013	6	Yes	Yes	3
Basel zoo	*Pongo abelii*	November 2013	6	Yes	Yes	1
Leipzig zoo	*Pongo abelii*	March 2014	10	Yes	Yes	2

Supplementary Table S2: List of all subjects participating in the cognitive tasks.

Subject	Species	Age at testing	Current Zoo	# Zoos lived in	Sex
Ito	*Pongo pygmaeus*	6	Allwetterzoo Münster	1	Male
Pongo	*Pongo pygmaeus*	15	Allwetterzoo Münster	2	Male
Amos	*Pongo pygmaeus*	13	Apenheul	2	Male
Radja	*Pongo pygmaeus*	52	Apenheul	3	Female
Silvia	*Pongo pygmaeus*	49	Apenheul	2	Female
Jose	*Pongo pygmaeus*	21	Apenheul	2	Female
Sandakan	*Pongo pygmaeus*	32	Apenheul	3	Female
Samboja	*Pongo pygmaeus*	9	Apenheul	1	Female
Willie	*Pongo pygmaeus*	12	Apenheul	1	Male
Batu	*Pongo pygmaeus*	14	Twycross zoo	2	Male
Summer	*Pongo pygmaeus*	13	Blackpool zoo	1	Female
Cherie	*Pongo pygmaeus*	18	Blackpool zoo	1	Female
Mali	*Pongo pygmaeus*	20	Paignton zoo	2	Female
Gambira	*Pongo pygmaeus*	18	Paignton zoo	1	Female
Anette	*Pongo abelii*	30	Durrell wildlife trust	3	Female
Jaya	*Pongo abelii*	9	Durrell wildlife trust	1	Male
Dagu	*Pongo abelii*	28	Durrell wildlife trust	5	Male
Dana	*Pongo abelii*	25	Durrell wildlife trust	3	Female
Gina	*Pongo abelii*	49	Durrell wildlife trust	1	Female
Budi	*Pongo abelii*	8	Basel zoo	2	Male
Maia	*Pongo abelii*	5	Basel zoo	2	Female
Kila	*Pongo abelii*	13	Basel zoo	2	Female
Vendel	*Pongo abelii*	13	Basel zoo	4	Male
Djamuna	*Pongo abelii*	13	Dortmund zoo	3	Female
Toba	*Pongo abelii*	18	Dortmund zoo	2	Female
Tao	*Pongo abelii*	8	Dortmund zoo	2	Female
Walter	*Pongo abelii*	23	Dortmund zoo	4	Male
Dokana	*Pongo abelii*	25	Leipzig zoo	4	Female
Padana	*Pongo abelii*	16	Leipzig zoo	1	Female
Pini	*Pongo abelii*	25	Leipzig zoo	1	Female
Raja	*Pongo abelii*	13	Leipzig zoo	1	Female
Tanah	*Pongo abelii*	5	Leipzig zoo	1	Female
Suaq	*Pongo abelii*	5	Leipzig zoo	1	Male

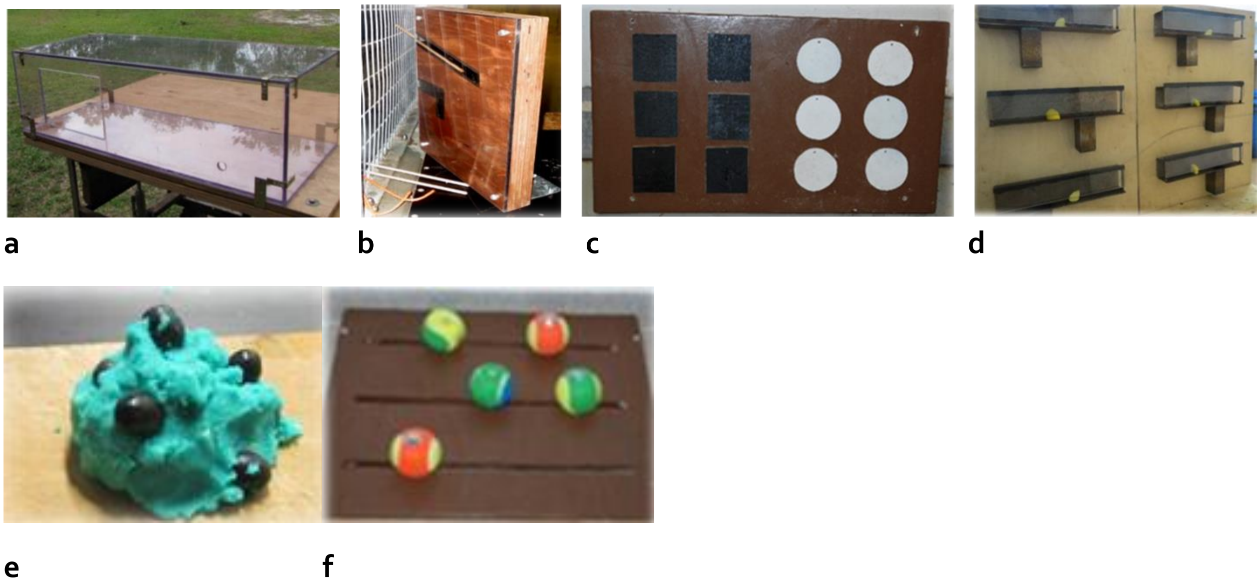

Supplementary Figure S1: Set of tasks for testing physical cognition and novelty response. Detour reaching task (**a**) The honey tool-task (**b**) Reversal learning board (**c**) Tube trap board (**d**) Novel Food (**e**) Novel toy (**f**).

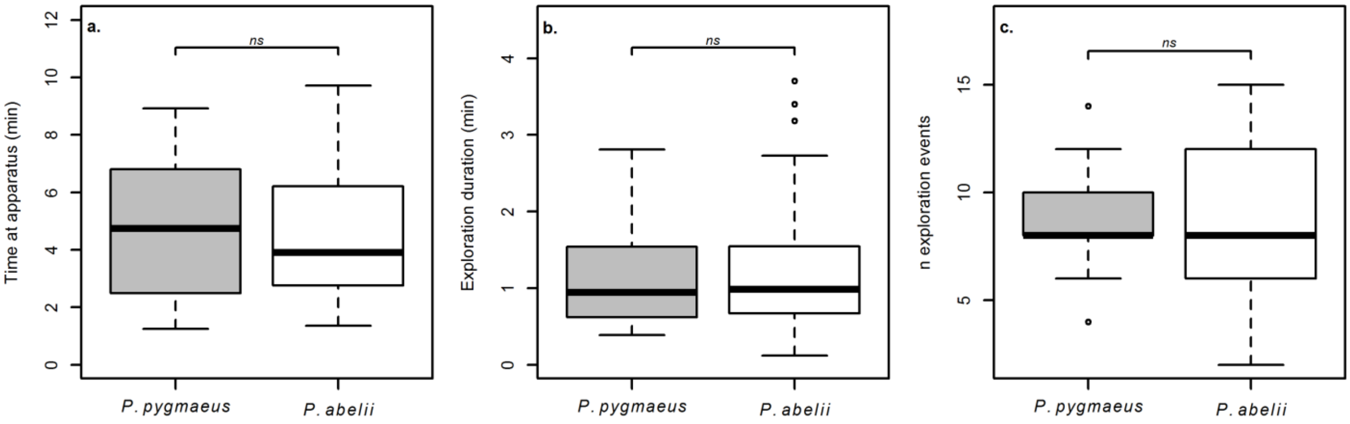

Supplementary Figure S2. Exploration behaviour: honey tool-task. Time spent at the honey tool-task (LM: $N_{Sumatra}$ =19, N_{Borneo} =13, $P_{species}$=0.903, P_{age}=0.064, P_{sex}=0.811) (**a**) and exploration duration during task participation (LM: $N_{Sumatra}$ =19, N_{Borneo} =13, $P_{species}$=0.398, P_{age}=0.094, P_{sex}=0.449) (**b**) as well as the variety of exploration acts used (LM: $N_{Sumatra}$ =19, N_{Borneo} =13, $P_{species}$=0.930, P_{age}=0.465, P_{sex}=0.523) (**c**).
